# Correction: Expression of genes with biomarker potential identified in skin from DSLD-affected horses increases with age

**DOI:** 10.1371/journal.pone.0326448

**Published:** 2025-06-16

**Authors:** Jennifer Hope Roberts, Jian Zhang, Florent David, Amy McLean, Karen Blumenshine, Eva Müller-Alander, Jaroslava Halper

Following the publication of this article [1], the authors identified errors in Figs 2 and 4. In Fig 2D, parameters unrelated to the data presented were mistakenly included; these have been removed. Furthermore, in both Figs 2D and 4, indicators of statistical significance were either missing or incorrectly applied. These have now been corrected, and the figures are consistent with the descriptions and interpretations provided in the published article. The corrected Figs 2 and 4 are provided here.

**Fig 2:**
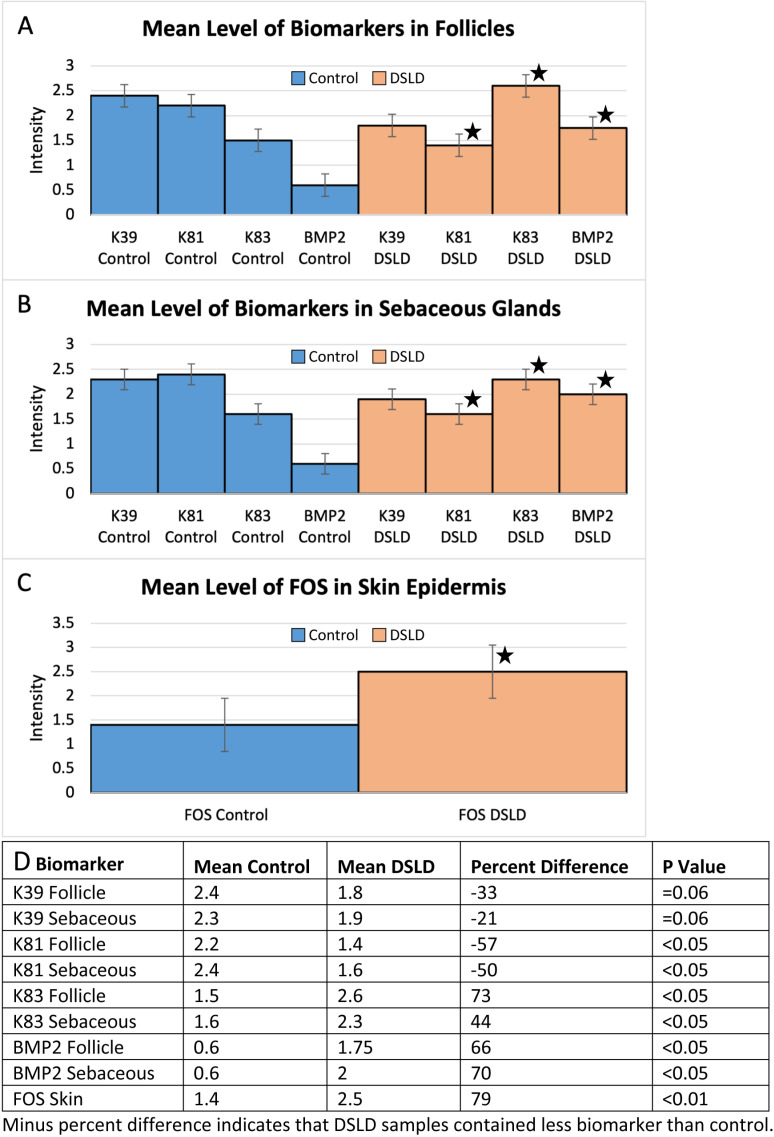
Summary of immunohistochemical staining for all markers. A. Mean staining of each biomarker in follicles. K39 control-mean 2.4, K39 DSLD-mean 1.8, p = 0.06; K81 control-mean 2.2, K81 DSLD = mean 1.4, p < 0.05; K83 control-mean 1.5, K83 DSLD-mean 2.6, p < 0.05; BMP2 control-mean 0.6, BMP2 DSLD-mean 1.75, p < 0.05. Star = highly significant difference between DSLD and corresponding control value. B. Mean staining of each biomarker in sebaceous glands. K39 control-mean 2.3, K39 DSLD-mean 1.9, p = 0.06; K81 control-mean 2.4, K81 DSLD-mean 1.6, p < 0.05; K83 control-mean 1.6, K83 DSLD-mean 2.3 p < 0.05; BMP2 control-mean 0.6, BMP2 DSLD-mean 2.0, p < 0.05. C. Mean of FOS in horses with DSLD compared with controls. Control-1.4 and DSLD- 2.5, p < 0.01. D. Immunohistochemistry staining parameters such as mean intensity, P-values, and percent differences between controls and DSLD cases or each biomarker. P values calculated using Wilcoxon rank sum test through JMP program.

**Fig 4:**
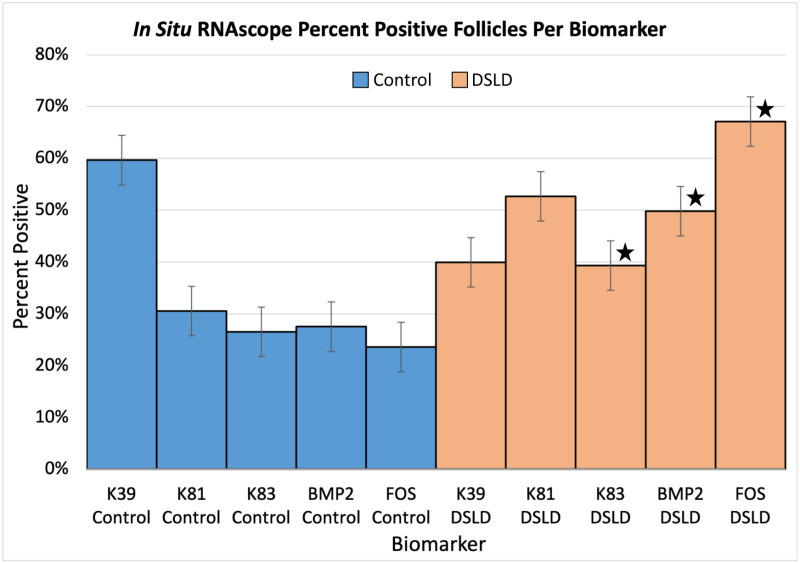
Percent positive follicles by biomarker for control and DSLD cases.

In addition, the primary data underlying the results reported in the published article [1] were not included with the original publication, despite the Data Availability Statement indicating that “All data are contained in Haythorn, A., et al., Differential gene expression in skin RNA of horses affected with degenerative suspensory ligament desmitis. *Journal of Orthopaedic Surgery and Research*, 2020; 15(1):460. https://www.ncbi.nlm.nih.gov/Traces/study/?acc=PRJNA544650.” With this Correction, the authors provide the complete underlying dataset as Supporting Information in S1 File. It can be viewed below.

The correct Data Availability statement for this article is: All relevant data are within the article and its Supporting Information files.

## Supporting information

S1 File Supplementary dataset. This file includes supplementary data.
(DOCX)
