# Supplementary material for: Expression of genes with biomarker potential identified in skin from DSLD-affected horses increases with age
Source: PLoS One. 2023 Jul 14;18(7):e0287740. doi: 10.1371/journal.pone.0287740 (PMC10348567; doi:10.1371/journal.pone.0287740)
Supplement: Attachment — Submitted filename: Response to Reviewers.docx [file pone.0287740.s001.docx]

May 29, 2023

RE: PONE-D-23-01721

Dear Dr. Sundaram:

Thank you and the reviewer with providing us with constructive comments. We addressed them to the best of our knowledge.

Concerning certain Journal requirements, we did provide information regarding animal treatment (in the Supporting Information section). The study was approved by institutional IACUC (line 108 in Material and Methods) Details about submission of supporting data from a previously done RNA-seq (Haythorn et al., 2020) can be found in an NIH repository at <https://www.ncbi.nlm.nih.gov/Traces/study/?acc=PRJNA544650>. Yes, the paper by Haythorn et al was published, see ref. number 3 in the manuscript. It is not a dual publication to this manuscript under review as it reports RNA -seq and identification of differentially expressed genes in RNA-seq. There was no attempt to establish any diagnostic criteria or assays using those data in the paper by Haythorn et al. The URL for the NCBI entry is now included in Data Availability section and in the manuscript itself (line 91).

The phrase “data not shown” was omitted. The reference list is complete and up to date.

Response to Editor comments: Statistical test methods and exact p-values are now included in the Result section and/or Figure legends where appropriate. Statistical evaluation was not done for results in Figure 3 because as we noted in the text the number of horses in each age group was limited (lines 365 and 366). We do not have scale bars on Fig. 5 (immunohistochemistry), but it is stated in the Figure legend that all photos are taken at 200x magnification. We did put together a Graphic abstract.

Response to Reviewer’s comments:

No. 3: <https://www.ncbi.nlm.nih.gov/Traces/study/?acc=PRJNA544650> is included in the body of the paper in the Introduction (line 91), and in Data Availability at the end of the manuscript. : Statistical test methods and exact p-values are included in the Result section and/or Figure legends.

No. 5: NGS was changed to RNA-seq throughout the manuscript. Equine geneticists are more interested in SNP analysis where breeds do play a role. Because we do not know whether DSLD is actually a single entity, and because Peruvian Paso horses suffer from higher prevalence of DSLD we cannot exclude the possibility that breed plays a role. The breed importance is comparable to the role of ethnicity and race background in human disease.

Introduction: We did add that in addition to Peruvian Pasos we were able to obtain skin biopsies from several other breeds, e,g, Arabians, Warmbloods and Hanoverians (lines 70 and 71). As far as we know no other RNA-seq study has been done so far (line 87).

Material and methods: A reference for 2^-∆∆Ct^ method using glyceraldehyde-3-phosphate dehydrogenase (GAPDH) and actin beta (ACTB) was added (ref 6) as was one more reference gene (actin beta): line 146, Tables 3 and 4).

Results: standard deviations and statistical differences were marked in appropriate figures.

Discussion: yes, RNA-seq was performed on RNA isolated from skin samples from control and DSLD horses (lines 472 and 473), and also see in Introduction, lines 87 and 88.

I hope that I was able to answer all questions and queries to your satisfaction.

Your sincerely,

Jaroslava Halper, MD, PhD

Department of Pathology

College of Veterinary Medicine

The University of Georgia

and Department of Basic Science

AU/UGA Medical Partnership

Athens, GA USA
