# Supplementary material for: Correction: Expression of genes with biomarker potential identified in skin from DSLD-affected horses increases with age
Source: PLoS One. 2025 Jun 16;20(6):e0326448. doi: 10.1371/journal.pone.0326448 (PMC12169534; doi:10.1371/journal.pone.0326448)
Supplement: S1 File — This file includes supplementary data. (DOCX) [file pone.0326448.s001.docx]

**Statistics**

A two-sided T-test as well as the Wilcoxon rank sum test were used to compare the results of

immunohistochemistry and RNAscope assays for each biomarker along with the mean, standard

deviation, and percent difference between control and DSLD affected horses (JMP Pro

2022 Statistical Program). We used a *p* value of less than 0.05 to determine statistical significance,

and biomarkers determined significant through these tests were established as the best

representatives for a panel that may be used to diagnose DSLD. For non-statistically significant

results (P*>*0.05), a power analysis was conducted (α = 0.05, two-sided T-test) to determine the

risk of committing Type II errors design.

Using JMP Pro 16, receiver operating characteristic (ROC) curves graphically displaying

the trade-off between sensitivity and specificity were generated for different cut-off points. For

the diagnostic assay to adequately distinguish between normal/controls (X axis) and diseased/

DSLD horses /Y axis), only ROC curves with an area under the curve (AUC) of 0.7 or higher

were considered as considered. We selected our cut off value using Youden’s Index. We also

considered one value above and one value below Youden’s Index to include varying levels of

sensitivity and specificity.

**Table 5**. **Immunohistochemistry data.**  NSA= no sample available.

| **Description** | **Sex** | **Age** | **Breed** | **K39 IHC (Fol)** | **K39 IHC (Seb)** | **K81 IHC (Fol)** | **K81 IHC (Seb)** | **K83 IHC (Fol)** | **K83 IHC (Seb)** | **BMP2 IHC (Fol)** | **BMP2 IHC (Seb)** | **FOS IHC** |
| --- | --- | --- | --- | --- | --- | --- | --- | --- | --- | --- | --- | --- |
| **Control 1** | M | 24 | Tenn Walking | 3 | 3 | 3 | 3 | 3 | 3 | 0.5 | 2 | 0 |
| **Control 2** | M | 13 | Appaloosa | 3 | 3 | 2.5 | 3 | 1 | 1 | 1 | 1 | 3 |
| **Control 3** | M | 9 | Oldenberg | 3 | 1 | 2.5 | 3 | 1 | 1 | 1.5 | 2 | 1 |
| **Control 4** | M | 27 | Thoroughbred | 2 | 2 | 2.5 | 3 | 3 | 4 | 1.5 | 2 | 0 |
| **Control 5** | F | 5 | Quarter | 3 | 3 | 3 | 2.5 | 0 | 1 | 1 | 1 | 2 |
| **Control 6** | M | n/a | Quarter | 2 | 3 | 2 | 2 | 2 | 2 | 0 | 0 | 2 |
| **Control 7** | M | n/a | Quarter | 2 | 1 | 2.5 | 3 | 3 | 4 | 0 | 0 | 0 |
| **Control 8** | M | n/a | Quarter | 2 | 2 | 2 | 2 | 0 | 0 | 2 | 1 | 2 |
| **Control 9** | F | 26 | Peruvian Paso | 3 | 3 | 0 | 0 | 4 | 0 | 0 | 0 | 2.5 |
| **Control 10** | F | 8 | Peruvian Paso | 2 | 3 | 2 | 2 | 0 | 1 | 0 | 0 | 2.5 |
| **Control 11** | F | 8 | Peruvian Paso | 2 | 2 | 1 | 0 | 1 | 1 | 0 | 0 | 1 |
| **Control 12** | F | 6 | Peruvian Paso | 2 | 2 | 0 | 1 | 1 | 2 | 0 | 0 | 3 |
| **Control 13** | n/a | n/a | Arabian | 3 | 3 | 1 | 2 | 1.5 | 1.5 | 1 | 1 | 2 |
| **Control 14** | n/a | n/a | Arabian | 2 | 1 | 0 | 0 | 1 | 2 | 0 | 0 | 0 |
| **DSLD 1** | F | 7 | Peruvian Paso | 2 | 1 | 0 | 1 | 3 | 3 | 2 | 1 | 2.5 |
| **DSLD 2** | M | 13 | Gray Tenn Walk | 2 | 2 | 2.5 | 3 | 0 | 1 | 3 | 3 | 1 |
| **DSLD 3** | M | 13 | Peruvian Paso | 1.5 | 3 | 0 | 0 | 0 | 4 | 0 | 2 | 4 |
| **DSLD 4** | M | 8 | Peruvian Paso | 3 | 3 | 0 | 0 | 0 | 1 | 0 | 0 | 3 |
| **DSLD 5** | N/A | N/A | Peruvian Paso | 2 | 2 | 0 | 0 | 1 | 3 | 0.5 | 0 | 2.5 |
| **DSLD 6** | M | 12 | Andalusian | 3 | 2 | 3 | 3 | 0 | 1 | 1 | 2 | 2 |
| **DSLD 7** | F | 14 | German Riding Pony | 2 | 2 | 3 | 3 | 3 | 3 | 0 | 0 | NSA |
| **DSLD 8** | F | 16 | Arabian | 1 | 1 | 1 | 3 | 2 | 1 | 2 | 2 | NSA |
| **DSLD 9** | F | 10 | Arabian | 3 | 3 | 3 | 3 | 1 | 1 | 1 | 2 | 3 |
| **DSLD 10** | F | 11 | Arabian | 2 | 2 | 3 | 3 | 2 | 0 | 0 | 2 | 3 |
| **DSLD 11** | M | 6 | Peruvian Paso | 2 | 1 | 2.5 | 3 | 0 | 0 | 3 | 3 | 3 |
| **DSLD 12** | M | 20 | Quarter | 3 | 3 | 3 | 3 | 2 | 3 | 3 | 3 | 3 |
| **DSLD 15** | M | 27 | Painted TB | 2 | 2 | 2 | 3 | 3 | 3 | 0 | 0 | 1.5 |
| **DSLD 16** | M | 10 | Spanish Breed | 1 | 2 | 0 | 0 | 3 | 3 | 0 | 0 | 1.5 |
| **DSLD 17** | M | 14 | Zweibrucker | 3 | 2 | 3 | 3 | 3 | 3 | 2 | 2 | 2 |
| **DSLD 18** | F | 17 | Arabian | 2 | 1 | 2 | 1 | 1 | 1 | 1.5 | 1.5 | 3 |
| **DSLD 19** | F | n/a | Arabian | 1.5 | 2 | 1 | 1 | 3 | 3 | 1 | 1 | 2 |
| **DSLD 20** | F | n/a | Arabian | 2 | 3 | 1 | 1 | 3 | 2 | 0 | 0 | 0 |
| **DSLD 21** | F | n/a | Arabian | 2 | 2 | 2 | 2 | 3 | 3 | 1 | 1 | 2 |
| **DSLD 22** | M | 15 | German WB | 0.5 | 0 | 1 | 1 | 3 | 3 | 0 | 0 | 0 |
| **DSLD 23** | M | 11 | Thoroughbred | 1 | 1.5 | 0 | 0 | 3 | 1 | 0 | 0 | 3 |
| **DSLD 24** | M | 10 | Peruvian Paso | 0.5 | 0 | 0 | 0 | 4 | 4 | 0 | 0 | 0 |
| **DSLD 25** | F | 26 | Thoroughbred | 2 | 2 | 0 | 0 | 3 | 3 | 0 | 0 | 3 |

**Table 6. RNAscope Data.** Scoring for each biomarker (except for *FOS* expression in epidermis) was done by percentage. *FOS* epidermis score was based on intensity 0= none present and +++= high intensity. N/A= not available.

| **RNAscope Control** | | | | | | |  | **RNAscope DSLD** | | | | | | |
| --- | --- | --- | --- | --- | --- | --- | --- | --- | --- | --- | --- | --- | --- | --- |
| **Case** | ***K39*** | ***K81*** | ***K83*** | ***BMP2*** | ***FOS*** | ***FOS* Epidermis** |  | **Case** | ***K39*** | ***K81*** | ***K83*** | ***BMP2*** | ***FOS*** | ***FOS* Epidermis** |
| **Control 1** | 76 | 0 | 56 | 0 | 0 | 0 |  | **DSLD 1** | 30 | 10 | 0 | 50 | 100 | +++ |
| **Control 2** | 28 | 100 | 8 | 0 | 0 | 0 |  | **DSLD 2** | 0 | 20 | 0 | 20 | 100 | 0 |
| **Control 3** | 100 | 10 | 0 | 0 | 0 | 0 |  | **DSLD 3** | 25 | 0 | 14 | 12 | 0 | 0 |
| **Control 4** | 0 | 0 | 36 | 29 | 0 | 0 |  | **DSLD 4** | N/A | N/A | 100 | 80 | 80 | +++ |
| **Control 5** | 100 | 10 | 58 | 0 | 100 | 0 |  | **DSLD 5** | 100 | 100 | 80 | 73 | 0 | +++ |
| **Control 6** | 71 | 30 | 36 | 80 | 30 | +++ |  | **DSLD 6** | 67 | 100 | 20 | 0 | 0 | ++ |
| **Control 7** | 60 | 30 | 10 | 34 | 100 | +++ |  | **DSLD 7** | 20 | 0 | 75 | 0 | 0 | + |
| **Control 8** | 100 | 0 | 32 | 60 | 50 | 0 |  | **DSLD 8** | 0 | 20 | 0 | 50 | 0 | 0 |
| **Control 9** | 100 | 0 | 0 | 0 | 0 | 0 |  | **DSLD 9** | 0 | 100 | 59 | 0 | 0 | ++ |
| **Control 10** | 0 | 20 | 25 | 0 | 50 | ++ |  | **DSLD 10** | 60 | 0 | 10 | 80 | 100 | +++ |
| **Control 11** | 0 | 67 | 10 | 0 | 0 | 0 |  | **DSLD 11** | 100 | 0 | 0 | 0 | 100 | + |
| **Control 12** | 100 | 30 | 50 | 10 | 0 | 0 |  | **DSLD 12** | 0 | 50 | 77 | 100 | 95 | ++ |
| **Control 13** | 10 | 80 | 30 | 20 | 0 | 0 |  | **DSLD 15** | 0 | 100 | 0 | 100 | 100 | +++ |
| **Control 14** | 90 | 50 | 20 | 50 | 0 | 0 |  | **DSLD 16** | 50 | 80 | 100 | 100 | N/A | +++ |
|  |  |  |  |  |  |  |  | **DSLD 17** | 100 | 100 | 76 | 75 | 100 | +++ |
|  |  |  |  |  |  |  |  | **DSLD 18** | 50 | N/A | 10 | 100 | 100 | +++ |
|  |  |  |  |  |  |  |  | **DSLD 19** | 100 | 100 | 100 | 50 | 0 | ++ |
|  |  |  |  |  |  |  |  | **DSLD 20** | 76 | 100 | 95 | 86 | 100 | +++ |
|  |  |  |  |  |  |  |  | **DSLD 21** | 0 | 100 | 0 | 100 | 100 | 0 |
|  |  |  |  |  |  |  |  | **DSLD 22** | 10 | 10 | 0 | 0 | 100 | 0 |
|  |  |  |  |  |  |  |  | **DSLD 23** | 10 | 100 | 70 | 0 | 100 | ++ |
|  |  |  |  |  |  |  |  | **DSLD 24** | 80 | 100 | 30 | 10 | 0 | ++ |
|  |  |  |  |  |  |  |  | **DSLD 25** | 75 | 80 | 50 | 0 | 0 | +++ |
|  |  |  |  |  |  |  |  | **DSLD 26** | 100 | 0 | 100 | 100 | 100 | +++ |
